# Temporal-spatial cross attention network for recognizing imagined characters

**DOI:** 10.1038/s41598-024-59263-5

**Published:** 2024-07-04

**Authors:** Mingyue Xu, Wenhui Zhou, Xingfa Shen, Junping Qiu, Dingrui Li

**Affiliations:** 1 College of Information Engineering, Zhejiang University of Water Resources and Electric Power, Hangzhou, 310018 Zhejiang China; 2 Department of Computer Science and Technology, Hangzhou Dianzi University, Hangzhou, 310018 Zhejiang China

**Keywords:** Biological techniques, Bioinformatics

## Abstract

Previous research has primarily employed deep learning models such as Convolutional Neural Networks (CNNs), and Recurrent Neural Networks (RNNs) for decoding imagined character signals. These approaches have treated the temporal and spatial features of the signals in a sequential, parallel, or single-feature manner. However, there has been limited research on the cross-relationships between temporal and spatial features, despite the inherent association between channels and sampling points in Brain-Computer Interface (BCI) signal acquisition, which holds significant information about brain activity. To address the limited research on the relationships between temporal and spatial features, we proposed a Temporal-Spatial Cross-Attention Network model, named TSCA-Net. The TSCA-Net is comprised of four modules: the Temporal Feature (TF), the Spatial Feature (SF), the Temporal-Spatial Cross (TSCross), and the Classifier. The TF combines LSTM and Transformer to extract temporal features from BCI signals, while the SF captures spatial features. The TSCross is introduced to learn the correlations between the temporal and spatial features. The Classifier predicts the label of BCI data based on its characteristics. We validated the TSCA-Net model using publicly available datasets of handwritten characters, which recorded the spiking activity from two micro-electrode arrays (MEAs). The results showed that our proposed TSCA-Net outperformed other comparison models (EEG-Net, EEG-TCNet, S3T, GRU, LSTM, R-Transformer, and ViT) in terms of accuracy, precision, recall, and F1 score, achieving 92.66$\%$, 92.77$\%$, 92.70$\%$, and 92.58$\%$, respectively. The TSCA-Net model demonstrated a 3.65$\%$ to 7.49$\%$ improvement in accuracy over the comparison models.

## Introduction

BCIs can restore communication for paralyzed patients who have lost their ability to move or speak. Imagery handwriting is the focus of BCI research, where participants only write letters in mind without performing the actual action^1,2^. BCI allows users to communicate with external devices efficiently by interpreting the neural signals of imagined characters. The imagery-based handwriting paradigm is widely utilized in the BCI fields, where neural information generated by internally imagined actions is translated into control signals. There are two main strategies in deep learning for decoding imagined character tasks: CNN-based and RNN-based^3–7^.

CNNs have an advantage in perceiving local information. Pei et al.^5^ first applied independent component analysis to decompose the Electroencephalogram (EEG) signals and extract features for training a CNN-based classifier to recognize handwritten characters. Ullah et al.^3^ introduced a system that utilized a deep convolutional neural network to recognize visual/mental imagery of English alphabets, enabling direct typing through brain signals. However, CNNs have limitations in processing temporal features of neural signals due to the fixed size of the convolution kernel^6,7^.

RNNs exhibit advantages in processing sequence data, such as text and time series^8–13^. Willett et al.^14^ introduced a brain-to-text communication method by utilizing neural signals from the motor cortex and decoding them using a recurrent neural network. Sun et al.^15^ proposed a Brain2Char architecture that decodes character sequences from electrocorticography (ECoG) signals using 3D Inception layers, bidirectional recurrent layers, and dilated convolution layers. However, RNNs encounter gradient vanishing or exploding when processing long data sequences^16^.

We argue that CNN and RNN decoding techniques restrict capturing the global context information of neural signals. Moreover, there is an overlap between the temporal and spatial characteristics of neural signals during activities^17^. However, these overlaps cannot be fully grasped using CNN and RNN approaches.

The Transformer is a deep neural network that utilizes the self-attention mechanism to effectively capture long dependencies in sequential data^18–21^. It has been used for neural signal decoding, which makes use of its ability to capture temporal information along the time series^22,23^. Furthermore, cross-attention is a technique employed in neural networks to identify intricate connections between multiple sequences or multimodal data^24^. It is deployed in the Transformer, which allows one sequence to attend to another, establishing dependencies between them. Inspired by the cross-attention^24^, we introduced a Temporal-Spatial Cross Attention Network (TSCA-Net) in decoding imagined characters. The contributions of our paper are as follows:

- We cascaded LSTM and Transformer in a Temporal Feature (TF) module to extract temporal features from MEA signals. By harnessing the benefits of LSTM’s gating mechanism, we capture continuous temporal features within the time dimension of MEA signals. Subsequently, we employ the Transformer to capture the global dependencies among distinct temporal sequences.
- We introduced the Transformer into the Spatial Feature (SF) module for the extraction of spatial features. It allowed the Transformer to capture long-term dependencies in the channel sequence, enabling the extraction of spatial features from the MEA signals.
- We developed a Temporal-Spatial Cross (TSCross) module that comprises two essential sub-modules, namely TSCross-SingleT and TSCross-SingleC. It is designed to extract the interplay between temporal and spatial features.

The organization of this paper is as follows. In the section “Introduction”, we present a concise overview of previous studies that have employed deep learning models for feature extraction from character signals. The section “Methods” introduced the overall structure of TSCA-Net and provided details about its key components. In the section “Experiments”, we conducted experiments to compare the classification accuracy of TSCA-Net with other models. Additionally, we conducted ablation experiments to assess the importance of each component. Finally, we summarized the contributions of this paper and identified future research directions.

**Figure 1:**
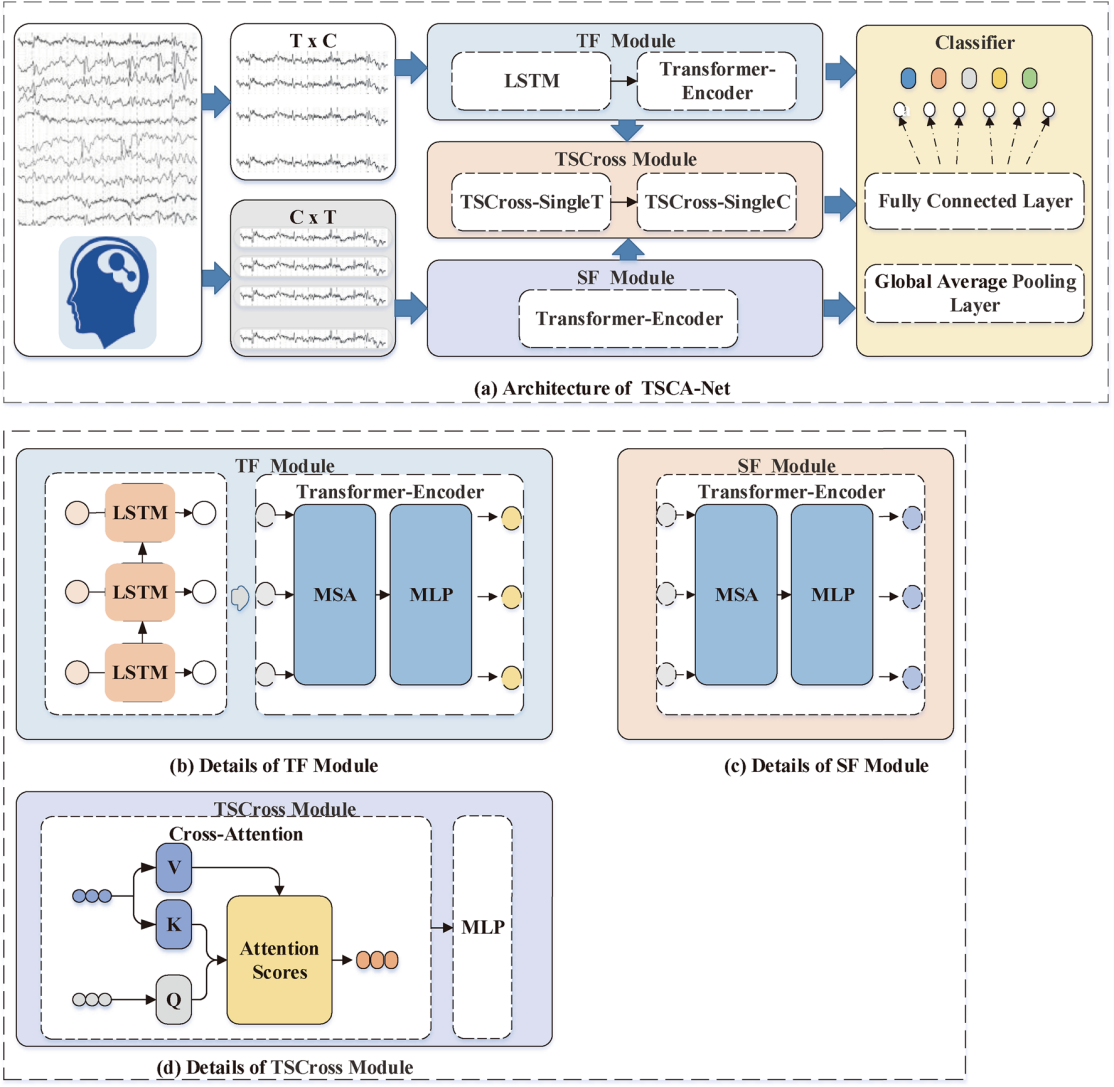
The overall architecture of TSCA-Net. (**a**) TSCA-Net consists of four modules: TF, SF, TSCross, and Classifier, as illustrated. (**b**) The TF module combines cascaded LSTM and Transformer models to extract temporal features from MEA signals. (**c**) In the SF module, the Transformer model is employed to capture long-term dependencies among the channel sequences. (**d**) The TSCross module leverages a cross-attention mechanism to capture the interdependencies of both temporal and spatial features. It comprises two submodules, namely TSCross-SingleT and TSCross-SingleC. The Classifier module is utilised to predict the category labels of input MEA signals, consisting of a global pooling layer and two fully connected layers. Notably, the encoder of the Transformer model is exclusively deployed within the TSCA-Net framework.

## Methods

To effectively capture the temporal and spatial features of imagined character signals, we developed the TSCA-Net model, which consists of four modules: TF, SF, TSCross, and Classifier, as shown in Fig. 1. The detailed procedures for each module in TSCA-Net are described below.

### Overall architecture

The TF module was implemented to capture long-term temporal dependencies in neural signals. The time series of signals were initially inputted into the LSTM module, which is responsible for capturing continuous temporal features. Subsequently, the processed temporal representation was fed into the transformer module, which extracted the inter-dependencies among different positions by calculating attention scores^25^.

The SF module was designed to capture the spatial relationships between different channels on a global scale. In the SF procedure, the channel series of the signals are directly inputted into the transformer module, which computes attention scores to capture the inter-dependencies. Unlike the TF, the SF utilizes the raw channel sequences as input.

The TSCross module captures the correlation between temporal and spatial features of neural signals. Due to the distinct dependency relationship between time and channels, the TSCross module was divided into two sub-modules: TSCross-SingleT and TSCross-SingleC. TSCross-SingleT computed the attention degree of acquisition channels at different time points, while TSCross-SingleC computed the attention degree of different time points on the acquisition channels.

In the TSCross-SingleT processing flow, both the channel sequences of the neural signals and the representation obtained by the TF were fed into the TSCross. The channel sequences were encoded as the query vector to calculate the attention scores, while the representation obtained was encoded as the key and value vectors for attention calculation.

Similarly, in the TSCross-SingleC processing flow, the time sequence and the representation captured by the SF were used as inputs to the TSCross. The time sequence was encoded as the query vector for attention calculation, while the obtained representation was encoded as the key and value vectors.

The Classifier is a crucial component of the TSCA-Net, responsible for mapping the features of input data to corresponding category labels or probability distributions. It takes the fused representation captured by TF, SF, and TSCross as its input. The Classifier plays a vital role in the TSCA-Net by assigning category labels or probability distributions to input data features. It takes the combined representation captured by TF, SF, and TSCross as its input. The processed features go through two fully connected layers before being fed into the softmax function. The softmax function function generates a probability distribution for each possible classification. The predicted label is determined by selecting the category with the highest probability.

### Multi-head self-attention

The Multi-head Self-Attention (MSA) mechanism allows for focusing on multiple positions in a sequence at the same time^26^. This is achieved by mapping individual elements of the input sequence to three vectors: key, value, and query. The mechanism then calculates the output by taking a weighted sum of these vectors. The MSA is made up of several heads, each with its own set of key, value, and query vectors. These vectors are used to attend to different pieces of information within the sequence. Finally, the output vectors of each head are combined through concatenation or weighted averaging to produce the final output.

1 $$\begin{aligned} &\text {MSA}(Q, K, V)={\text {concat}}\left( \text {head}_1, \ldots , \text {head}_{N_h}\right) W^o\\&\text {head}_i=\text {Attention}\left( Q W_i^Q, K W_i^K, V W_i^V\right) \\&\text {Attention}\left( Q_i, K_i, V_i\right) ={\text {Softmax}}\left( \frac{Q_i K_i^\text { T}}{\sqrt{d_k}}\right) V_i \end{aligned}$$

The MSA formula is shown in Eq. (1), where $\text {head}_i$ refers to the *i*-th attention head out of a total of $N_h$ heads. The linear transformation matrix for mapping the concatenated inputs to the output space is denoted as $W^o\in {\mathbb {R}}^{h d_{v}\times d_{\text {model}}}$, where $d_{\text {model}}$ represents the dimension of input embedding. The query, key, and value vectors of the *i*-th head are represented by $Q W_i^Q$, $K W_i^K$ and $V W_i^V$ respectively, where $W_i^Q\in {\mathbb {R}}^{d_{\text {model}}\times d_q}$, $W_i^K\in {\mathbb {R}}^{d_{\text {model}}\times d_k}$, and $W_i^V\in {\mathbb {R}}^{d_{\text {model}}\times d_v}$ are the corresponding weight matrices, and $d_q$, $d_k$ and $d_v$ are dimensions of the query, key, and value vectors.

### Relative position embedding

The traditional Transformers have relatively weak position relations within the input sequences^26–28^. Therefore, it requires a position embedding to enhance its positional awareness when using a Transformer for modelling. The ViT model introduced a learnable absolute position embedding that adds a fixed vector to each position within the input sequence^18^. However, this method is unable to depict the relative relationships between positions accurately.

To enhance the location perception of the Transformer, TSCA-Net deploys relative positions embedding to generate the vector representations of each other dynamically^29^. Specifically, the relative position information in TSCA-Net is represented by a two-dimensional matrix with dimensions equal to the length of the input sequence. During the attention computation, the position biases indexed via the relative position matrix are added to the attention scores. The enhanced MSA is represented by Equation (1), where the weight of the vector position is denoted by *B*.

2 $$\begin{aligned} \text {Attention}\left( Q,K,V\right) =\text {SoftMax}\left( QK^\text {T}/\sqrt{d}+B\right) V, \end{aligned}$$

### Cross-entropy loss function

The cross-entropy function is a type of convex function that helps prevent the model from getting stuck in suboptimal solutions. During the backpropagation, the model parameters are updated continuously using gradient information from the loss function^30^. The detailed formula is defined as Eq. (3), where $N_t$ represents the number of trials and $N_c$ represents the number of categories. $y_{m}^{n}$ and ${\hat{y}}_m^n$ refer to the ground-truth labels and the predicted labels for the *m*th trial, respectively.

3 $$\begin{aligned} {\mathscr {L}}=-\frac{1}{N_t} \sum _{n=1}^{N_c} \sum _{m=1}^{N_t} y_{m}^{n} \log \left( {{\hat{y}}_m^n}\right) , \end{aligned}$$

## Experiments

### Data acquisition and preprocessing

To evaluate the effectiveness of our proposed model, we conducted experiments on the Imagery Handwritten Character dataset^14^. The dataset records the neural activity of one participant from two microelectrode arrays placed in the hand area of the precentral gyrus over 10 days. The participant had a high-level spinal cord injury and was paralyzed from the neck down, with hand movements limited to twitching and micromotion. He was instructed to imagine holding a pen and writing letters and symbols by hand on ruled paper, as if not paralyzed.

The raw handwriting-imagination dataset comprises 26 English letters and five special characters (commas, apostrophes, question marks, periods, and spaces). These special characters serve as pauses or cues during the imagination handwriting. After a time-warping step was applied, as described in the literature^14^, the five special characters were removed from the dataset. Subsequently, by merging the data collected over ten days, we obtained a collection of 3,172 trials of spiking activities, encompassing 26 English lowercase letters. Each trial sample was represented as a two-dimensional matrix with 201 time steps and 192 electrodes. In our experiments, we aimed to train the model to classify 26 lowercase letters.

### Implementation details of the TSCA-Net

To explain the TSCA-Net employed in the experiments, we presented detailed information about its key modules: TF, SF, TSCross, and Classifier. Specific implementations and parameters are listed in Table 1.

**Table 1:** Details of the TSCA-Net Architecture. In the table, *C* represents the number of MEA channels, *T* represents the number of time points, and *N* represents the number of output classes.

Modules	Layers	Output size	Details
TF	Input Size: $\textit{T}\times \textit{C}$		
	BN	$\textit{T}\times 256$	Batch Normalization
	LSTM	$\textit{T}\times 256$	[$inputdim=256, hiddendim=256$] $\times 1$
	BN	$\textit{T}\times 256$	Batch Normalization
	MSA	$\textit{T}\times 2048$	[$heads=16,d_q=d_k=d_v=128$]
	MLP	$\textit{T}\times 256$	2 FC layers [2048,256]
SF	Input Size: $\textit{C}\times \textit{T}$		
	BN	$\textit{C} \times 256$	Batch Normalization
	MSA	$\textit{C} \times 2048$	[$heads=16,d_q=d_k=d_v=128$]
	MLP	$\textit{C} \times 256$	2 FC layers [2048,256]
TSCross-SingleT	Query Size: $\textit{T}\times 256$,		Time Vectors as Query
	Key, Value Size: $\textit{C} \times \textit{T}$		Channel Vectors as Key
	MSA	$\textit{C} \times 2048$	[$heads=16,d_q=d_k=d_v=128$]
	MLP	$\textit{C} \times 256$	2 FC layers [2048,256]
TSCross-SingleC	Query Size: $\textit{C}\times 256$,		Time Vectors as Query
	Key, Value Size: $\textit{T} \times \textit{C}$		Channel Vectors as Key
	MSA	$\textit{T} \times 2048$	[$heads=16,d_q=d_k=d_v=128$]
	MLP	$\textit{T} \times 256$	2 FC layers [2048,256]
Classifier	Input1 Size: $\textit{C}\times 256$		The TSCross-SingleT output
	Input2 Size: $\textit{T} \times 256$		The TSCross-SingleC output
	Concatenate		Concatenate the input vectors
	AvgPooling	$1\times 256$	Global average pooling
	MLP	$1\times \textit{N}$	FC-1 layers [256, 256],
			FC-2 layers [256, *N*],softmax

### Platform environment

Our experiments were conducted on a Linux operating system with the TITAN XP GPU. Our model was implemented with PyTorch 1.10.2 and Python 3.7.12. We utilized the Adam optimizer with a weight decay of 0.0001 and an initial learning rate of 3e-5, and trained our model for 400 epochs with a batch size of 8.

### Metrics

To evaluate the performance of our proposed model, we used four metrics: accuracy, precision, recall, and F1 scores. Accuracy is the proportion of correctly classified samples, while precision measures the proportion of true positive samples among all samples classified as positive. Similarly, recall measures the proportion of true positive samples among all actual positive samples. The F1 score is a balanced measure of the model’s performance and represents the harmonic mean of precision and recall. The mathematical formulas for calculating these metrics are shown in Eqs. (4), (5), (6), and (7). Due to the limited size of the experimental dataset, we conducted a five-fold cross-validation in the experiment. Specifically, we split the dataset into training and testing sets in a 4:1 ratio. The evaluation metrics were performed on the testing set for each of the five folds, and the average of these five evaluations was used to assess the model’s performance.

4 $$\begin{aligned} {\textrm{Accuracy}}= \frac{TP+TN}{TP+FN+TN+FP}\times 100\%, \end{aligned}$$

5 $$\begin{aligned} {\textrm{Precision}}= \frac{TP}{TP+FP}\times 100\%, \end{aligned}$$

6 $$\begin{aligned} {\textrm{Recall}} = \frac{TP}{TP+FN}\times 100\%, \end{aligned}$$

7 $$\begin{aligned} {\textrm{F1}} = \frac{2\times {\textrm{Precision}} \times {\textrm{Recall}}}{{\textrm{Precision}}+{\textrm{Recall}}}\times 100\%. \end{aligned}$$

where *TP* is the true positive, *FN* is the false negative, *TN* is the true negative, and *FP* is the false positive.

### Comparison experiments

We conducted comparison experiments with seven commonly classification models including EEG-Net^31^, EEG-TCNet^32^, GRU^14^, LSTM^33^, S3T^22^, R-Transformer^34^, and ViT^18^ To evaluate the performance of TSCA-Net in decoding signals for classification tasks.

- EEG-Net and EEG-TCNet are convolution-based models that utilize CNNs or spatiotemporal convolutional neural networks to extract temporal and spatial features from EEG signals.
- GRU and LSTM are recurrent neural networks used for time series analysis and sequence modeling.
- S3T, R-Transformer, and ViT are three models of the Transformer type. S3T, a Transformer-based model developed by South China University of Technology, is designed to model spatiotemporal features of EEG signals. R-Transformer developed by Michigan State University combines RNNs and multi-head attention. ViT, a vision transformer developed by Google, addresses long-range dependency in image classification.

**Table 2:** Performance comparison of TSCA-Net with seven state-of-the-art models.

Model	Accuracy (%)	Precision (%)	Recall (%)	F1 (%)
EEG-Net^31^	85.24	88.70	87.65	88.17
EEG-TCNet^32^	85.17	85.76	84.86	85.04
GRU^14^	86.70	84.57	83.36	83.96
LSTM^33^	89.01	90.02	88.92	89.47
S3T^22^	88.50	89.10	88.45	88.77
R-Transformer^34^	86.08	87.80	87.93	87.86
ViT^18^	85.62	85.88	85.83	85.40
TSCA-Net (Ours)	92.66	92.77	92.70	92.58

After conducting five experiments in cross-validation, the results of eight different models were evaluated in terms of accuracy, precision, recall, and F1 score. The results are presented in Table 2. TSCA-Net demonstrated the highest performance with accuracy, precision, recall, and F1 score values of 92.66$\%$, 92.77$\%$, 92.70$\%$, and 92.58$\%$, respectively. It was found that EEG-TCNet had the weakest performance among all the compared models. Among the two CNN models, EEG-Net performed slightly better than EEG-TCNet, possibly because of its simpler architecture and ease of training and optimization. Regarding the two RNN models, it was found that LSTM exhibited superior classification performance over GRU. ViT had the lowest performance among the Transformer models, trailing behind the other three models. However, EEG-TCNet achieved the highest accuracy among the CNN models, while ViT had the lowest accuracy among the Transformer models. These findings suggest that Transformer models are more effective than CNN models for recognizing imagined characters.

**Table 3:** Cross-validation results of comparative models.

Model	Fold-1	Fold-2	Fold-3	Fold-4	Fold-5	Mean	Std.
EEG-Net^31^	85.31	86.80	85.09	83.82	85.23	85.24	1.06
EEG-TCNet^32^	85.33	86.83	85.10	83.48	85.10	85.17	1.19
GRU^14^	84.50	86.47	88.20	87.59	86.73	86.70	1.41
LSTM^33^	88.91	87.63	90.36	87.88	90.25	89.01	1.28
S3T^22^	87.32	91.79	87.98	86.74	88.65	88.50	1.98
R-Transformer^34^	85.92	83.86	86.23	86.55	87.82	86.08	1.43
ViT^18^	85.76	85.92	85.59	88.71	82.12	85.62	2.34
TSCA-Net (Ours)	**94.30**	**92.56**	**92.41**	**91.77**	**92.25**	**92.66**	**0.96**

Significant values are in bold.

**Figure 2:**
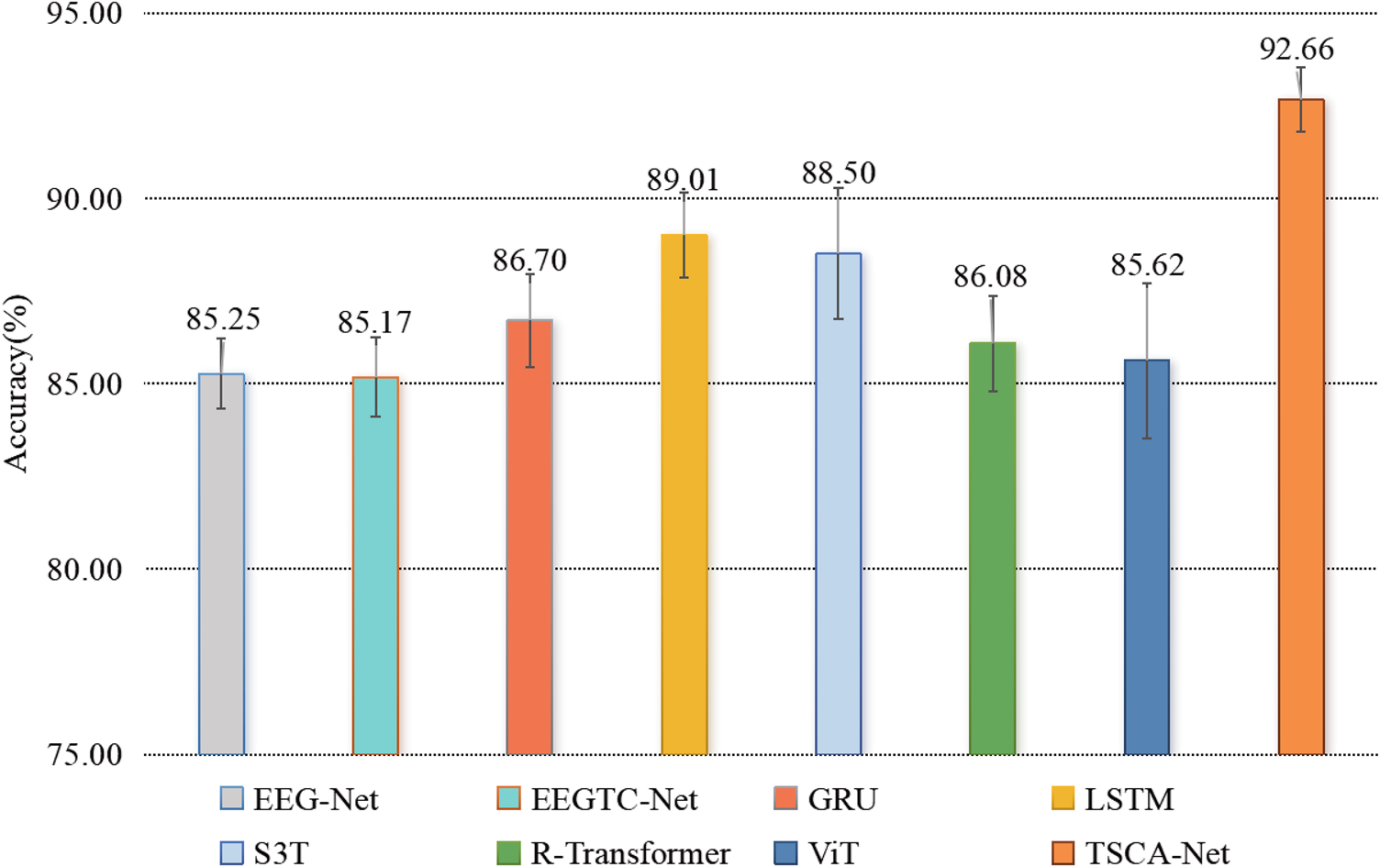
Statistics of standard deviation and mean of accuracy.

The statistical results of comparative models obtained through five-fold cross-validation experiments are shown in Table 3. TSCA-Net achieved the highest mean accuracy and the lowest standard deviation among all five experiments. Moreover, Fig. 2 demonstrates that the lowest accuracy of TSCA-Net is still better than the optimal performance of other comparison models. Overall, TSCA-Net has consistently performed well and outperformed the other comparison models.

**Figure 3:**
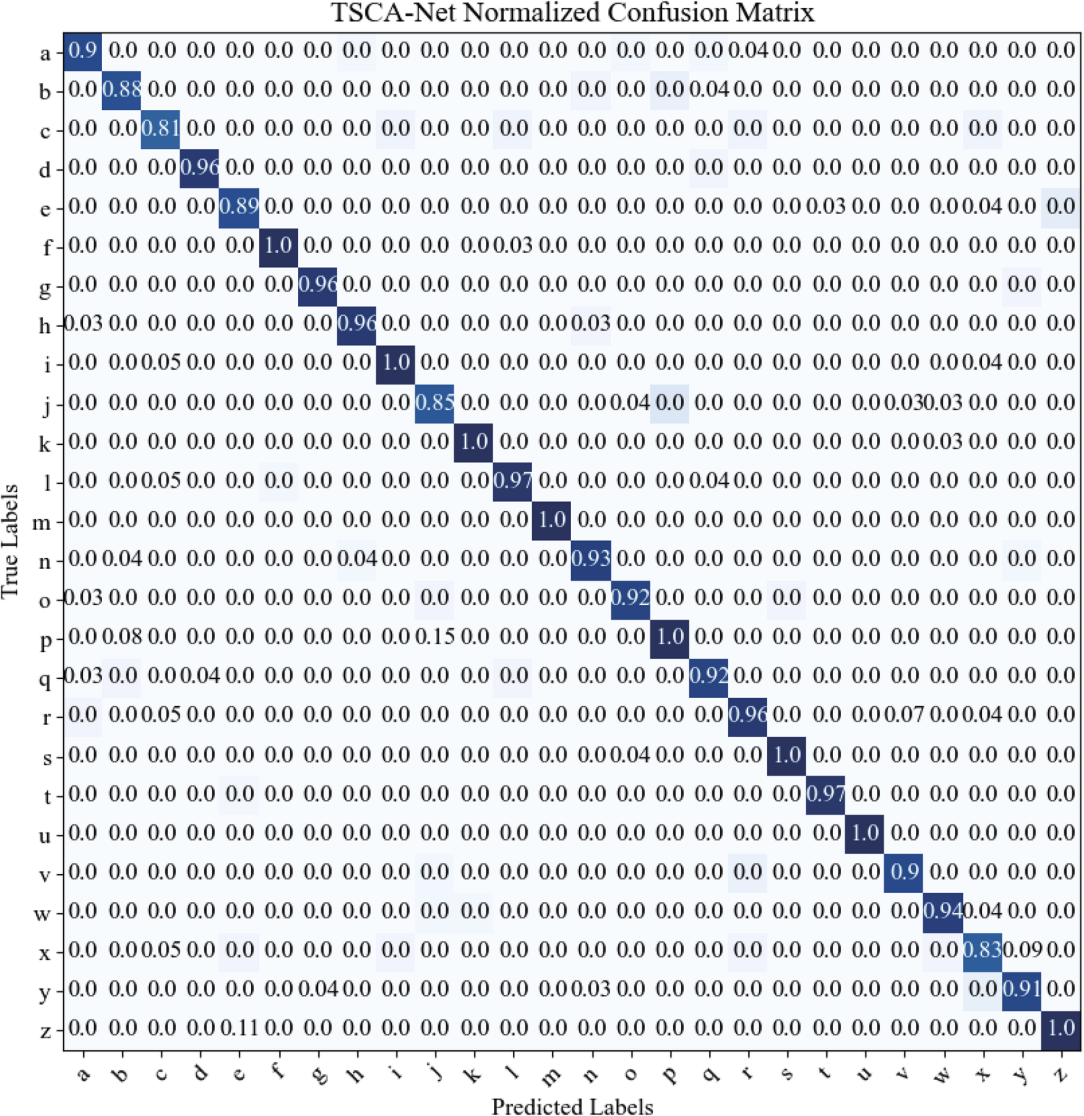
Confusion matrix. The horizontal axis represents the predicted categories by the TSCA-Net, and the vertical axis represents the true labels of the 26 lowercase letters.

**Table 4:** The precision and recall of TSCA-Net for recognizing 26 characters.

Letter	Precision (%)	Recall (%)	F1 (%)	Letter	Precision (%)	Recall (%)	F1 (%)
a	89.70	96.30	92.88	n	93.10	93.10	93.10
b	88.50	95.80	92.01	o	92.30	96.00	94.11
c	81.00	100.00	89.50	p	100.00	93.30	96.53
d	96.00	100.00	97.96	q	91.70	91.70	91.70
e	88.90	92.30	90.57	r	95.80	85.20	90.19
f	100.00	95.80	97.85	s	100.00	95.20	97.54
g	95.80	100.00	97.85	t	96.90	100.00	98.43
h	95.80	92.00	93.86	u	**100.00**	**100.00**	**100.00**
i	100.00	88.90	94.12	v	90.00	100.00	94.74
j	100.00	88.00	93.62	w	93.50	96.70	95.07
k	100.00	95.50	97.70	x	82.60	86.40	84.46
l	96.60	93.30	94.92	y	90.90	90.90	90.90
m	**100.00**	**100.00**	**100.00**	z	100.00	86.40	92.70

Significant values are in bold.

**Figure 4:**
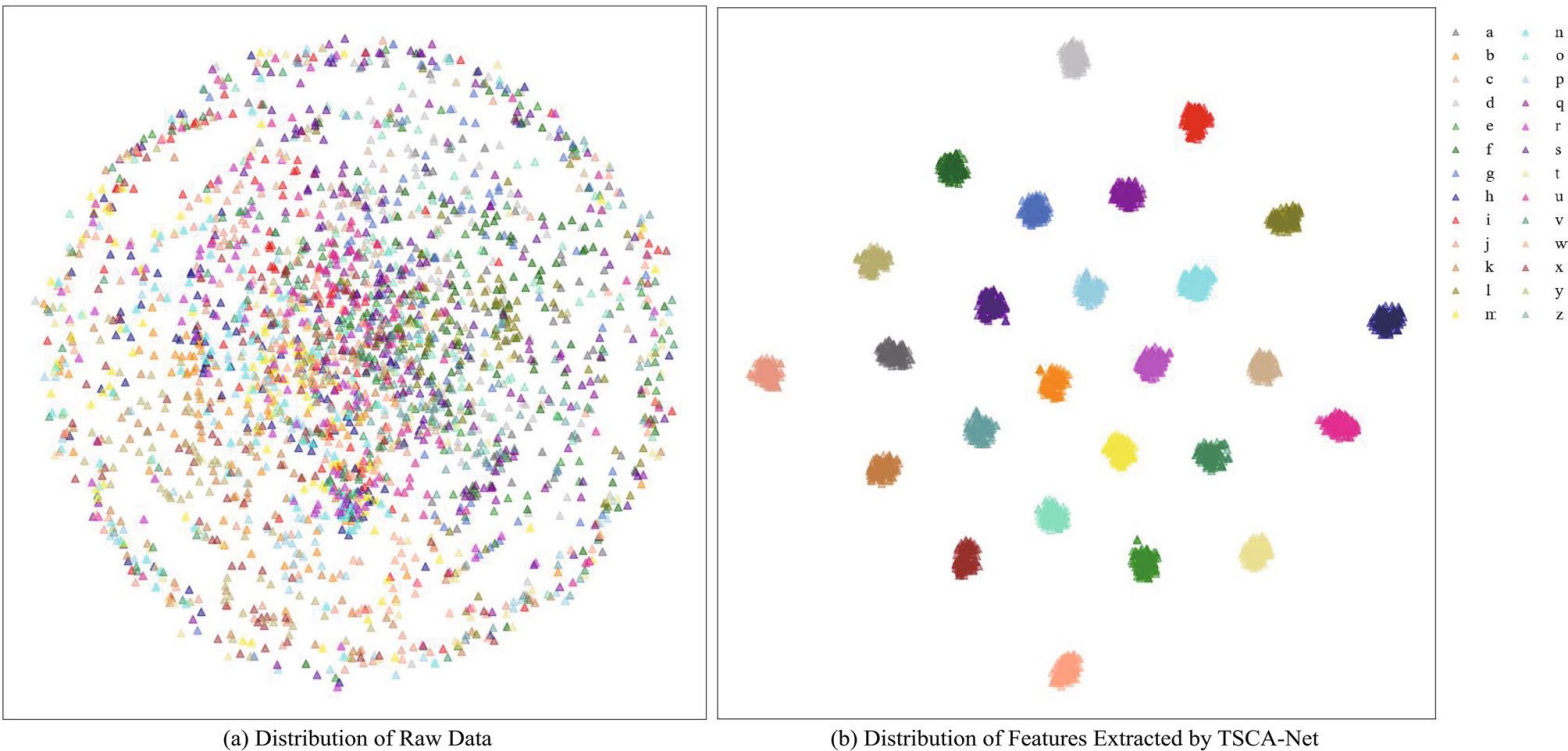
Visualization of data distribution using t-SNE.

From the confusion matrix presented in Fig. 3, we can see that the diagonal elements have the darkest colors. It indicates that TSCA-Net has relatively low error rates for identifying imagery handwritten characters. The model has achieved a remarkable 100$\%$ precision and recall rates for recognizing characters such as ’m’ and ’u’.

To further validate the performance of TSCA-Net, we conducted a comparative analysis of the distribution of the raw characters data and the distribution of extracted with TSCA-Net. In Fig. 4a, the distribution of the 26 letters in the raw dataset appears highly chaotic. Characters belonging to the same category cannot be effectively clustered in a 2D space and exhibit a random distribution. However, in Fig. 4b, it can be observed that after the feature extraction process by TSCA-Net, the 26 letters are distributed into 26 distinct clusters. The letters are tightly clustered within the same group, and the boundaries between groups are distinguishable by different categories.

Table 4 shows the optimal TSCA-Net model used for character signals decoding, which achieved an accuracy rate of 94.30$\%$. The table highlights that the TSCA-Net model delivered exceptional precision and recall rates in predicting characters ’m’ and ’u’, which had a 100$\%$ rate. However, the precision for character ’c’ prediction was the lowest among all the characters, and the recall for character ’r’ prediction was also the lowest. These findings suggest that the proposed TSCA-Net model is a robust and reliable method for decoding MEA signals in imagery handwriting tasks, and it outperforms other models.

## Ablation experiments

In the ablation experiments, we analyzed the effect of the TSCA-Net model performance based on three factors: model components, position embedding, and core parameters of multi-head attention.

### Ablation experiments of model components

In our experiments evaluating TSCA-Net, we analyzed the impact of three components: TF, SF, and TSCross. Results are presented in Table 5. Our findings show that the absence of the TF has the most significant impact on the accuracy of the TSCA-Net model. The accuracy of the model decreased by 5.69$\%$ compared to the optimal model. Similarly, the absence of the SF component resulted in a decrease of 2.44$\%$ in accuracy, with the TSCA-Net accuracy dropping to 91.86$\%$.

**Table 5:** Ablation study of TSCA-Net components. The effects of LSTM and Transformer in TF, SingleT, and SingleC in TSCross, and SF modules on the performance of the TSCA-Net model were explored. Here SingleT represents TSCross-SingleT, SingleC represents TSCross-SingleC.

TF		SF	TSCross		Accuracy (%)
LSTM	Transformer		SingleT	SingleC	
$\times$	$\checkmark$	$\checkmark$	$\checkmark$	$\checkmark$	88.79
$\checkmark$	$\times$	$\checkmark$	$\checkmark$	$\checkmark$	88.61
$\checkmark$	$\checkmark$	$\times$	$\checkmark$	$\checkmark$	91.86
$\checkmark$	$\checkmark$	$\checkmark$	$\times$	$\times$	89.87
$\checkmark$	$\checkmark$	$\checkmark$	$\checkmark$	$\times$	92.41
$\checkmark$	$\checkmark$	$\checkmark$	$\times$	$\checkmark$	91.45
$\checkmark$	$\checkmark$	$\checkmark$	$\checkmark$	$\checkmark$	**94.30**

Significant values are in bold.

As a part of our study, we analyzed the effect of TSCross-SingleT and TSCross-SingleC components on accuracy, both individually and combined. We observed that the removal of both components resulted in a decrease in accuracy by 4.43$\%$. However, when only TSCross-SingleT was removed, the accuracy decreased by 2.85$\%$, whereas the removal of only TSCross-SingleC caused a decrease of 1.89$\%$.

The experiments suggest that temporal features have the most significant impact on character recognition accuracy, followed by the spatiotemporal features acquired through the spatiotemporal interaction module. Conversely, the impact of spatial features is relatively negligible. We found that using spatial channels instead of time as a query in the MSA of the TSCross has a relatively significant impact on character recognition accuracy.

**Table 6:** Ablation study of position embedding. Absolute, Sin/Cos, Relative, and None are four distinct position embedding methods, where None indicates the absence of position embedding in the model.

Position embedding				Accuracy (%)
Absolute	Sin/Cos	None	Relative	
$\checkmark$				91.30
	$\checkmark$			90.95
		$\checkmark$		91.74
			$\checkmark$	**94.30**

Significant values are in bold.

### Ablation experiments of position embedding

In the ablation experiments, we assessed the accuracy of four position embedding methods: relative, absolute, Sin/Cos, and none. From the experimental results presented in Table 6, it can be concluded that the TSCA-Net accuracy is highest using relative position embedding, while Sin/Cos position embedding produces the lowest accuracy. Specifically, the accuracy with relative position embedding is 2.56$\%$ higher than without position embedding, 3.00$\%$ higher than with absolute position embedding, and 3.35$\%$ higher than with Sin/Cos position embedding. These findings suggest that relative position embedding is the most effective approach to improve the accuracy of TSCA-Net in character recognition tasks.

**Table 7:** Ablation study of the multi-head self-attention with different hyperparameters. Heads and Dims represent the number of attention heads and the embedding dimension, respectively.

Heads	Dims	Accuracy (%)	Heads	Dims	Accuracy (%)
8	64	91.58	16	64	92.06
8	128	91.83	16	128	**94.30**
8	256	92.22	16	256	93.19

Significant values are in bold.

### Ablation experiments of the core parameters of the MSA

We performed experiments to determine the core parameters of multi-head attention in the TSCA-Net model. Specifically, we varied the number of heads and the encoding dimension. Based on the results presented in Table 7, we found that setting the number of heads to 16 and the encoding dimension to 128 led to the highest character recognition accuracy of 94.30$\%$. Therefore, we set these values as the default for the TSCA-Net model.

## Conclusion

This paper proposed TSCA-Net, a spatiotemporal cross-attention network for recognizing imagined characters. TSCA-Net consisted of four modules: TF, SF, TSCross, and Classifier. The TF module combined LSTM and Transformer to capture the temporal features of neural signals, while the SF module focused on capturing spatial information. The TSCross module was introduced to learn the relationship between the temporal and spatial features.

Experimental results on the Imagery Handwritten Character dataset demonstrated that TSCA-Net outperformed other comparison models in accuracy, precision, recall, and F1 score. Additionally, TSCA-Net provided an effective approach for extracting MEA signal features in brain-computer interface systems.

However, we manually selected the hyperparameters of TSCA-Net for cross-validation based on recognition accuracy. The absence of nested cross-validation for hyperparameter selection will lead to a potential risk of overfitting. This challenge needs to be addressed in future research efforts.

## Data Availability

The handwriting BCI dataset in this study is publicly available on GitHub at https://github.com/xy21yue/imagined-character-data.

## References

[1] Lotte F (2018). A review of classification algorithms for EEG-based brain-computer interfaces: A 10 year update. J. Neural Eng..

[2] Guillot A, Moschberger K, Collet C (2013). Coupling movement with imagery as a new perspective for motor imagery practice. Behav. Brain Funct..

[3] Ullah S, Halim Z (2021). Imagined character recognition through EEG signals using deep convolutional neural network. Med. Biol. Eng. Comput..

[4] Janapati R, Desai U, Kulkarni SA, Tayal S (2023). Human-Machine Interface Technology Advancements and Applications.

[5] Pei L, Ouyang G (2021). Online recognition of handwritten characters from scalp-recorded brain activities during handwriting. J. Neural Eng..

[6] Han K (2020). A survey on vision transformer. IEEE Trans. Pattern Anal. Mach. Intell..

[7] Lecun Y, Bengio Y, Hinton G (2015). Deep learning. Nature.

[8] Sutskever I, Vinyals O, Le QV (2014). Sequence to sequence learning with neural networks. Adv. Neural Inf. Process. Syst..

[9] Lotte F, Congedo M, Lécuyer A, Lamarche F (2007). A review of classification algorithms for EEG-based brain-computer interfaces. J. Neural Eng..

[10] Ma, X., Qiu, S., Du, C., Xing, J. & He, H. Improving EEG-based motor imagery classification via spatial and temporal recurrent neural networks. In *2018 40th Annual International Conference of the IEEE Engineering in Medicine and Biology Society (EMBC)*, 1903–1906 (IEEE, 2018). 10.1109/EMBC.2018.8512590 30440769

[11] Alhagry S, Fahmy AA, El-Khoribi RA (2017). Emotion recognition based on EEG using LSTM recurrent neural network. Int. J. Adv. Comput. Sci. Appl..

[12] Dai, Z. *et al.* Transformer-xl: Language modeling with longer-term dependency. ICLR 2019 (2018).

[13] Beltagy, I. Peters, M. E. & Cohan, A. Longformer: The long-document transformer. arXiv preprint arXiv:2004.05150 (2020).

[14] Willett FR, Avansino DT, Hochberg LR, Henderson JM, Shenoy KV (2021). High-performance brain-to-text communication via handwriting. Nature.

[15] Sun P, Anumanchipalli GK, Chang EF (2020). Brain2char: A deep architecture for decoding text from brain recordings. J. Neural Eng..

[16] Pascanu, R., Mikolov, T. & Bengio, Y. On the difficulty of training recurrent neural networks. JMLR.org (2012).

[17] Gordon, S. M., Jaswa, M., Solon, A. J. & Lawhern, V. J. Real world bci: cross-domain learning and practical applications. In *Proceedings of the 2017 ACM Workshop on an Application-oriented Approach to BCI out of the Laboratory*, 25–28 ( 2017).

[18] Dosovitskiy, A., Beyer, L., Kolesnikov, A., Weissenborn, D. & Houlsby, N. An image is worth $$16\times 16$$ words: Transformers for image recognition at scale. arXiv preprint arXiv:2010.11929 (2020).

[19] Dai, Z., Yang, Z., Yang, Y., Carbonell, J. & Salakhutdinov, R. Transformer-xl: Attentive language models beyond a fixed-length context. arXiv preprint arXiv:1901.02860 (2019).

[20] Wen, Q. *et al.* Transformers in time series: A survey. arXiv preprint arXiv:2202.07125 (2022).

[21] Zhou, D. *et al.* Refiner: Refining self-attention for vision transformers. arXiv preprint arXiv:2106.03714 ( 2021).

[22] Song, Y., Jia, X., Yang, L. & Xie, L. Transformer-based spatial-temporal feature learning for EEG decoding. arXiv preprint arXiv:2106.11170 ( 2021).

[23] Tibrewal N , Leeuwis N, Alimardani M (2022). Classification of motor imagery EEG using deep learning increases performance in inefficient BCI users. PLoS One.

[24] Chen, C. F., Fan, Q. & Panda, R. Crossvit: Cross-attention multi-scale vision transformer for image classification. In *Proceedings of the IEEE/CVF International Conference on Computer Vision* (2021).

[25] Han K (2021). Transformer in transformer. Adv. Neural Inf. Process. Syst..

[26] Vaswani, A. *et al.* Attention is all you need. arXiv (2017).

[27] Devlin, J., Chang, M. W., Lee, K. & Toutanova, K. Bert: Pre-training of deep bidirectional transformers for language understanding. arXiv preprint arXiv:1810.04805 (2018).

[28] Shaw, P., Uszkoreit, J. & Vaswani, A. Self-attention with relative position representations. arXiv preprint arXiv:1803.02155 (2018).

[29] Liu, Z. *et al.* Swin transformer: Hierarchical vision transformer using shifted windows. In *Proceedings of the IEEE/CVF International Conference on Computer Vision* (2021).

[30] He, K., Gkioxari, G., Dollar, P. & Girshick, R. Mask R-CNN. In *International Conference on Computer Vision* (2017).

[31] Lawhern VJ (2018). EEGNet: A compact convolutional network for EEG-based brain-computer interfaces. J. Neural Eng..

[32] Ingolfsson, T. M. *et al.* EEG-TCNet: An accurate temporal convolutional network for embedded motor-imagery brain–machine interfaces. In *2020 IEEE International Conference on Systems, Man, and Cybernetics (SMC)* 2958–2965 (IEEE, 2020).

[33] Raviprakash H (2020). Deep learning provides exceptional accuracy to ECoG-based functional language mapping for epilepsy surgery. Front. Neurosci..

[34] Wang, Z., Ma, Y., Liu, Z. & Tang, J. R-transformer: Recurrent neural network enhanced transformer. arXiv preprint arXiv:1907.05572 (2019).

